# Foreign

**Published:** 1841-08-28

**Authors:** 


### FOREIGN.

*Case of rupture of the Liver and of the Spleen.* By ALEXANDER KILGOUR, M. D.—A considerable number of cases of rupture of the liver and spleen may be found scattered through medical authors; but I do not think there is one where the accident was of the nature of the following case.

Case I. *Rupture of the Liver.*—Christopher Brown, aged 36, a shoemaker, was brought to the hospital on the evening of the 8th September, from the watch-house, with a note to the effect, that it was a case of inflammation of the bowels.

At the visit on the forenoon of the 9th, he was found complaining of severe pain over his bowels. The abdomen was very tense, tympanitic, and the least pressure gave great pain. His countenance was anxious and contracted; his skin not morbidly hot nor cold; his pulse 108, sharp, small, and wiry; his tongue brown and much furred. He had no vomiting, nor had he retched any. His bowels had now been open, he stated, for several days; had slept none during the night; and was constantly moaning and complaining of his bowels. The account given, according to the statement of the nurse, on inquiry from those who brought him in the police truck, was, that he had been picked up in a gutter in a state of complete intoxication on the previous evening, and been taken to the watch-house, and that he had complained of constant pain in the bowels, and inability of moving from the pain.

Sixteen ounces of blood were ordered to be drawn from the arm; and twelve leeches were directed to be applied to the abdomen, and afterwards emollient poultices. A spoonful of castor-oil was ordered to be taken every two hours, till the bowels should be freely moved.

10th. Died this morning at seven o'clock, having been sensible to the last.

*Inspection*, twelve hours after death. No external appearance of injuries. On the scalpel entering the abdomen bloody fluid gushed out. The fluid was collected, and amounted to ten pounds, (twelve ounces to the pound.) Some coagula were found, but almost the whole was dark fluid blood. On passing the hand into the pelvis, to examine whether the bladder had been ruptured, a body was taken out, which was at once seen to be the gall-bladder, and which was about half distended. It was now evident that the cause of death was the separation of the gall-bladder from the liver, and the consequent effusion of blood. On turning up the liver it exhibited a rough surface, corresponding to a similarly sized rough surface on the upper part of the anterior aspect of the gall-bladder, being the part where it is united to the liver. Coagula were adherent to the under surface of the liver, and to the transverse arch of the colon, and were also lying between the liver and duodenum. The texture of the liver was infiltrated with blood for about two inches in depth, and a fissure to the size of an inch in length, extended inward from the fossa of the gall-bladder, and was about half-an inch deep.

The gall-bladder was about half-filled with a mixture of bile and blood. The intestines were much inflamed, being in some parts of a deep-red colour, and in others dark blue.

The brain and viscera of the chest were healthy.

It may here be stated, that there were rumours soon afloat, that this man Brown had been struck by the watchman on the night that he was taken to the watch-house. The case was taken up by the proper authorities, and at the trial at the circuit Court of Aberdeen, evidence was laid that Brown had been struck, and that the watchman had jumped upon his belly with his knees. The prosecutor for the crown departed from the plea of murder, and a verdict of culpable homicide was brought in, on which the watchman was sentenced to banishment for life. It came out on the trial that there had been a collusion to conceal the nature of this case at the watch-house, or, at any rate, such irregularities in regard to reporting it in the proper quarter, as to call for a severe reprimand from the judge.

On looking through the remarks that have been made by morbid anatomists on the subject of ruptures of the liver and spleen, there will be found some points that admit of a few comments, and which the above and following cases may serve to illustrate. Dr. Baillie says, "the liver is more liable to be ruptured by external violence than any other gland of the body, which probably arises from two causes. The one is, that in thin persons the liver, more especially when large, lies near the surface of the body, and therefore may be readily affected by a strong external pressure; the other is, that the liver consists of a structure, the parts of which are more easily separated from each other by pressure, than those of almost any other organ of the body. * * * When ruptures have taken place in the liver, they have happened from some strong pressure applied to the upper part of the abdomen, as, for instance, from the wheel of a carriage passing over that part of the body. Little pain has been felt from such an injury; which is a proof, among many others, of the liver not possessing much sensibility; and some of the persons to whom this accident has happened have lived for several days."—Morbid Anatomy, Lond. 1833, p. 196

II. Is the liver more liable to be ruptured by external violence than any other gland of the body? If we are to be strictly confined to "glands," Baillie is probably right; but there is little doubt he was thinking of a comparison with the other solid viscera in the abdomen. If so, the recorded cases would appear to give a preponderance of ruptures of the spleen; and other authorities are opposed to Baillie on this point. Platner says, and Morgagni who quotes him, seems to agree with him, "that rupture or laceration happens more frequently in the spleen than in any other viscus:

and the latter authority continues; "nor is this to be wondered at, if you attend to the soft substance thereof, and the thinness of the coat wherewith it is invested. But if it become softer by the force of disease, and, by a quantity of slowly circulated blood, is extended below the fortification of the ribs, it is much more easily ruptured; and pours out in less time the greater quantity of blood; and consequently brings on a more speedy death." Translat. by Alexander, Vol. iii. p. 212. But of this organ, Baillie says: "The spleen has sometimes been known to be ruptured in consequence of external pressure upon that side of the body where it is situated. When the spleen is of the ordinary size, an accident of this kind can very rarely take place, because it is well defended by the ribs of the left side; but when the spleen is very large, so that a part of it passes below the margin of the ribs into the cavity of the flank, such an accident may very rarely happen." Morbid Anatomy, p. 216. Baillie would appear not to have known much of the histories of cases of this kind; and his opinions, both in regard to rupture of the liver and of the spleen, seem to have been founded upon their comparative exposure to pressure or external injury. The faithfulness of Morgagni's statement as to the quantity of blood poured out, and the speedy dissolution, is supported by the following case that I saw, and with the notes of which I have been furnished by my colleague, Mr. Keith, in one of whose wards he was.

Case II.—*Rupture of the Spleen.* June 19th, 1838. James Sinclair, mason, aged 33, was brought into the hospital at 1 P. M., having been precipitated from a height of twenty feet along with the ruins of an old house, which he was engaged in pulling down. He was partly buried among the bricks and rubbish, and required to be dug out.

He was exceedingly faint; the surface of the body blanched and bloodless; the pulse almost gone at the wrist; he complained exceedingly of thirst; he laboured under great difficulty of breathing, and coughed up mucus mixed with blood; the belly was tense, full, and tender; several of the ribs were fractured. No effort could produce reaction, nor heat the body or limbs in the least. He continued sensible, however, to the last, and expired at half-past four, P. M., exactly four hours after the accident befel him.

Twenty-four hours after death the body was examined. Nine ribs were fractured at or near their angles; several had penetrated the substance of the lung on the left side. The abdomen was full to distension with effused blood, which had flowed from the vessels of the spleen, as this organ was found smashed into a perfect pulp, completely broken up and separated into small portions. The liver and other viscera in the abdomen were uninjured.

2. Baillie also thinks that "strong pressure" must be applied to the upper part of the abdomen, such as the wheel of a carriage; but in fact a blow of almost no magnitude may produce this effect, and it is probable that sudden and violent muscular contraction may produce the same. In the precognition (private judicial examination previous to committal of a prisoner for trial) a stick with a round knob or head, such as watchmen carry, was exhibited to me, and I was asked if the injury could have been caused by a blow with it; and I had no hesitation in saying that it might; and in this opinion Dr. Ogston concurred with me. Heister notices (Inst. Chir. p. i. lib. i. cap. 15. Not. a) a case of a boy who died from laceration of the abdominal viscera from being struck by his schoolmaster, "non nisi bacillo quodam tenuiori, sed paulo tamen vehementius." Pearson gives a case where the liver was almost entirely divided, where the young man fell from the sixth step of a ladder, striking the right hypochondrium and epigastrium on the edge of a pail which he carried in his hand. (Med. Trans. of the College of Physicians, Vol. iii. p. 378, Lond. 1785.) Abercrombie relates a case where a man, sitting carelessly on the edge of a cart, was thrown from it by a sudden jerk upon the road. He got up immediately, and scrambled into the cart, which was still in motion, and he did not appear to the person who was with him to have suffered any injury, but he soon became faint, and in a few minutes he was dead. On inspection the liver was found to be ruptured through a great part of the right lobe, and there was extensive hæmorrhage in the cavity of the abdomen." (Pathol. Researches on Stomach, &c., Ed. 1837, p. 355.) But it is not in the liver alone that we meet with rupture from such slight causes. Bohn relates a case where the spleen of a woman was ruptured so that she died in a short hour by a stroke on the left hppochondre from her husband, "baculo mediocris crassitiei;" and also another where extensive rupture of the same organ was produced by a cudgelling, (ex fustigatione,) whilst there was not the slightest discoloration (ne suggillatio quidem) of the integuments, (De Renunciat. Vulner. p. 383, Lips. 1689.) This circumstance, the absence of any external mark of injury, is carefully noted by the old writers as one of the most remarkable points connected with these cases; and in some seems to be the principal reason for recording them. In Brown there was no discolouration of the integuments, otherwise it would have been entered in the report. Heister records one where the liver was found entirely broken down from external violence, and yet no lesion was observed externally. (Loc. citat.)

3. Baillie states, that, "little pain has been felt from such an injury, which is a proof among many others of the liver not possessing much sensibility." Now, how stands the fact? Brown, according to the report, had very great pain; and on the trial, it came out, that he complained of the most agonizing pain from the moment he got the injury, and considered himself as a dead man. In Mr. Pearson's case, the pain is described as being excruciating from the beginning to the end. In a case of hæmorrhage from spontaneous rupture of the liver given by Sir Gilbert Blane, but which the history of the case would seem to connect with slight external injury, the patient was seized with severe pain in the left hypochondrium; and the left lobe of the liver was found on examination after death to be the seat of a rupture. (Trans. of a Soc. for the Improvement of Med. and Surg. Knowledge, Vol. ii. p. 18. Lond. 1800) Morgagni quotes a case from Lanzoni, where "one who was struck with a fist violently on the liver, and had in his belly an extravasation of blood and a rupture of the vessels in the liver, fell down upon the ground soon after receiving the blow, and expired in a miserable manner." Having neither Morgagni in the original, nor the authority to which he refers, I take Alexander's translation, as intimating that the patient died in much agony.

A case is given in the eighteenth volume of the Med. and Phys. Journal, of a man who was struck after he had taken a hearty meal, with the muzzle of a carronade, in the right hypochondrium. He fell down on the deck, complained of acute pain, and expired in about twenty minutes. There were no marks of violence externally; the abdomen was filled with blood, and a rupture of the right lobe of the liver extended to the *lobulus Spigelii*, disuniting the gall-bladder partially from its natural situation.

It was proven in the trial of the watchman, that Brown had been eating, and, more particularly, drinking freely before he was struck, which, from what occurred in this case, would seem to bring the gall bladder more within the influence of external injury.

In the case given by Dr. Abercrombie, there is no mention as to whether the patient felt much pain, but the speedy supervention of fainting might have prevented the pain. Andral relates a case of spontaneous rupture, where the patient, not previously exhibiting any symptoms of disease of the liver, on awakening one morning, felt rather ill, and complained of some pain in the abdomen. He expressed a wish to remain in bed, and was left alone, when after some hours he was found dead. (Clinique Medicale by Spillan, p. 899.) I am not wishing to call in question the opinion entertained by Baillie and others, as to the little sensibility of the liver generally, but merely to show that pain is a prominent symptom in rupture of that organ, whether that pain depends upon the injury of the liver itself, or on inflammation excited in the peritoneum and intestines.

4. Dr. Baillie states in conclusion, that some of the persons to whom this accident has happened have lived for several days. This quite accords with the existing histories of the several cases. Death is the result either of the loss of blood, the anæmia by effusion into the peritoneal cavity, or of inflammation in the serous membrane of the abdomen. Dr. Abercrombie's patient died almost instantly after the injury. Sir Gilbert Blane's case survived only five or six hours, the patient having fallen into a swoon, from which be never recovered. Mr. Pearson's lived only ten hours, the pulse not being perceptible from the first, and the extremities becoming colder and colder. Andral's patient also lived only a few hours. The following case, where I assisted at the dissection, is confirmatory of the rapidity with which death takes place, and also so far as to the pain attending such injuries.

Case III.—*Wound of the Liver.*—A man, name unknown, was found in the morning lying dead in a retired part of the Links, with a discharged pistol beside him. He had been seen from a rising ground in the vicinity, a short time before he was found dead, crawling about and writhing his body; but as it was supposed to be some person in a state of intoxcation who had slept there, no farther notice was taken of him; others, it was proven, had seen him late in the evening walking near the same place. When the body was examined, a wound was found in the right side over the region of the liver, and which had penetrated the right lobe in an oblique manner from before backwards. There had been no ball in the pistol, but the wadding was found imbedded at the bottom of the wound. The abdomen contained a large quantity of blood partly coagulated.

All these are examples where the individuals died from the immediate loss of blood. Brown lived, however, above three days, and his death was perhaps more attributable to inflammation than to loss of blood, notwithstanding the quantity found in the abdomen. Morgagni quotes from the *Commercium Literarium* an example of a man being violently struck by a horse in the region of the liver; so that the lower ribs were broken, and the gibbous part of that viscus was cleft with a great number of fissures, not very deeply, however, as I suppose, since the belly was not filled with blood, but with a great deal of bloody lymph: and the patient did not die before the fourth day. (Transl. Vol. iii. p. 214.) He also refers to a case by Hippocrates, where a boy received a kick from a mule on his belly and liver, and died on the fourth day. (Epid. I. v. n. 39, Not. n. 17, as Morgagni says.) But, as there was no dissection, Vallesius commenting on the case, thinks that the boy died from inflammation of these viscera, and not from rupture or the effusion of blood. Bohn quotes a case from Tulpius, where a wound of the liver, attended with hæmorrhage, was followed by death on the fortieth day, and on dissection a collection of pus was found in the liver at the seat of the wound. (De Renun. Vul. p. 368.)

5. Are there any symptoms by which we can discover rupture of the liver or spleen, where there is no perforating wound or external mark of injury? The only symptoms which afford a diagnosis from rupture of any of the hollow viscera appear to be those depending upon loss of blood; paleness of the countenance, fainting, indistinctness of the pulse, and rapid dissolution. There is the same acute pain attending rupture of the liver or spleen with effusion of blood, as rupture of the intestine and effusion of its contents. But in those cases where the individuals live for some time, have we any symptoms by which we may know that the liver is injured? Hennen says, "the usual symptoms which characterize these injuries are, yellowness of the skin and urine, derangement of the stomach and alimentary canal, and cutaneous affections, particularly great and distressing itching."—(Military Surgery, 2d ed. 430.)

Bohn also tells us (chiefly from Celsus, however,) that there is anxiety of the præcordium, faintings, pain in the neck and shoulders, vomiting, sometimes bilious, sometimes bloody, bloody stools, heat, and intense thirst. P. 364. In Brown there was neither yellowness of the skin, nor derangement of the stomach, nor bowels, nor vomiting. In those cases where there was an external wound, besides hæmorrhage in several instances by the wound itself, there was more or less of a discharge of bile, or of a bilious-colored glutinous fluid. Thomson and Hennen are at variance as to the existence of jaundice from wounds of the liver. (Report on Military Hospitals, &c. Edin. 1816, p. 101.) There are no symptoms by which we are likely to be able to distinguish rupture of the liver from rupture of the spleen. Some light may probably be thrown on the case by a history of the mode of injury. Strokes and blows on the right hypochondrium are most likely to injure the liver; whilst falls from a great height, or general compression of the body, as where an individual is buried under a fall of earth or part of a ruin, is most likely to be followed by rupture of the spleen. The recorded cases seem to show that rupture of the spleen is more speedily fatal than rupture of the liver, unless where the latter is very extensive; a circumstance which we might almost infer from the structure of the spleen.

There are very few examples collected of rupture of the gall-bladder without injury of the liver itself. There is a well-known case in the Med. Chir. Trans., Vol. iv. p. 330, of a stroke from theshaft a cart on the region of the liver, followed by bilious vomiting; where, three weeks after the injury, the belly, which was distended with fluid, was tapped on three different occasions, and forty-one ounces of what appeared to be pure bile taken away, the case terminating successfully. But the nature or composition of the fluid was not accurately examined, and it still remains a question whether this was bile or not. There is a case given by Thomson, however, where a collection of bile was found contained in a cavity formed by adventitious membrane, after a wound of the liver itself. Fallopius and Forestus hold wounds of the gall-bladder to be necessarily mortal, but not immediately so; whilst Helmont and others entertain the opinion that they are instantly fatal. Elliotson says, (Lectures by Rogers, p. 875,) "Now and then the gall-bladder has been ruptured. A woman came one day to St. Thomas's Hospital, and fell down dead; and, on opening her, the gall-bladder was found to have been suddenly ruptured. Death, I understood, took place instantly."

6. What will be the prognosis in rupture or wound of the liver? On this point authorities differ very much? Celsus places wounds of the porta of the liver as fatal, and wounds of the substance of the liver amongst those *quæ vix ad sanitatem perveniunt*, Lib. v. Cap. 26. Galen, commenting on one of the aphorisms, (6. Aph. 18, and not Aph. 15, as Bohn says,) seems to think, along with Hippocrates, that wounds of the liver are always fatal; at the same time that he was aware, that cases of cure, not only of wounds, but where part of the liver had been cut away, had been given. It would appear that, in the time of Galen, a distinction was made betwixt wounds of the substance and wounds of the vessels of the liver; the latter being considered mortal, the former, not so. But Bohn, strongly inclined to Galen's opinion, thinks there is here something like a distinction without a difference, and holds that at the utmost, a possibility of cure can only exist where the superficies of the organ is injured. It is by no means unusual in *post-mortem* examinations to meet with cicatrices, lines, or depressions, in the surface of the liver; and though it is probable that these, in the majority of cases, are the sequelæ of abscesses, they may be in some instances the result of ruptures of the substance of the organ. One of the most satisfactory cases of recovery after injury of the liver is one quoted from Hildanus in the Sepulchretum, p. 16-18. A man received a wound in the region of the liver, and when the surgeon proceeded to examine it, he found a piece of the liver projecting in the wound, which he seized with the forceps and cut off. Severe symptoms ensued, but he recovered entirely. Three years after, he died of continued fever; and, at the necropsy, they found a small portion of the lower part of the lobe wanting, and the wound *cicatrice eleganter obductum*. The experience of modern observers leads to the conclusion, that wounds and ruptures of the liver are not fatal, unless very extensive. "A deep wound of the liver," says Hennen, p. 429, "is as fatal as if the heart itself was engaged. The slighter wounds are recoverable, particularly if the membrane alone is injured." Thomson in his report, p. 98, says, "we saw twelve cases of wounds of the liver, in which considerable progress towards recovery had been made." So that the prognosis is not so entirely against the patient as the old writers supposed.—*Edinburgh Med. and Surg. Journ.*

---

Sir Charles Scudamore, we find, does not confine his treatment of phthisis and chronic bronchitis to the iodine inhalation alone, but very properly conjoins with it the internal use of the remedies which may be required. We have been asked our opinion as to the efficacy of this measure. In our practice we have used iodine largely internally, and occasionally by inhalation, in a very simple way, that is, merely placing some iodine in an open vessel, in a small room, and allowing the patient to remain there until the vapour became disagreeable; gradually, in this way, accustoming the lungs to iodine vapour largely diluted with atmospheric air. There may be, however, some advantages in resorting to a regular inhaler.

Is this method curative? From our experience in the use of iodine, as administered internally, we believe that it may be of great service as a direct application to a diseased mucous membrane—possibly to a cavity. It will irritate, to some extent, however, and thus may do harm; but the alterative action of the iodine is, in other respects, of service, and we should, therefore, be disposed to give it a fair trial. It promises most in those cases in which chronic bronchitis has preceded phthisis, and seems to be slowly passing into it.

The following is Dr. Scudamore's account of his invention:

The unreserved manner in which I have communicated the results of my experience in the treatment of consumption in your own Journal at different times, in the "Medical Gazette," Feb. 3, 1840, and in the book alluded to, should protect me from all suspicion of concealment, or of selfish conduct. You speak of a "recipé" as if the treatment of phthisis and chronic bronchitis consisted of doing one thing only. I recommend inhalation as forming a part of a system of treatment, but certainly a very important part; yet, in order to produce its good effects, the doses and the combinations of the several ingredients are always to be considered. The following is the formula of the iodine solution which I prescribe:—

℞. Iodine;
Iodide of potassium, of each, gr. vj.
Distilled water, ℥v ʒvj.;
Alcohol, ℥j.
Mix together, and use by inhalation.

I usually commence with a drachm of this mixture, proceeding gradually to the extent of half an ounce, (rarely more,) putting two-thirds the dose for the first half of the time (10, 15, or 20 minutes,) and the other third for the remainder, always adding thirty minims of a saturated tincture of conium, with an increase if the cough be very irritable. Occasionally I add some saturated tincture of ipecacuanha; and when the respiration is spasmodically affected, some ætherial tincture of the lobelia inflata. All this information, and a great deal more, I have before communicated.

I can truly declare that it is my earnest wish and constant study not only to increase my own information on the pathology and treatment of consumption, but, also, on all occasions to impart every information which I might hope to be useful and interesting to the whole profession.

I am happy to state that the young gentleman whose case I described in The Lancet, May 8, and whom I attended in consultation with Mr. Norton, of Dorset street, is entirely recovered. I have also recently been gratified with the almost restoration to health of a young man whose symptoms were of the most aggravated description; together with pectoriloquism unequivocally indicating a cavity, there were the most harrassing cough; foul puriform expectoration; very hurried respiration on the least exertion; rapid pulse; profuse night sweats; loss of sleep, sometimes for the whole night; great emaciation and excessive debility. In the first instance I quite despaired of the case. I can give the reference to the individual to any one who desires to have a proper curiosity satisfied. I am, Sir, your humble servant,

CHARLES SCUDAMORE.

P.S.—It is of great importance that the glass inhaler should be well fitted with capacious tubes, and that all the ingredients employed should be perfectly pure.—*London Lancet.*

Mr. Gray, a London Surgeon, claims the prior discovery of this combination; the iodine has often been used for inhalation, but not that we are aware of in combination with conium.

---

*Acetic Acid in Headach.* By ROBERT HOWARD, M. D.—Some years since I was induced to suppose that acetic acid, if properly administered, would prove an efficient remedy for common headach; and on making a trial of it in a severe case, which had previously existed many hours, it succeeded completely in a very short time. I have since had many opportunities of trying it in nervous headach; that arising from disordered stomach; headach following sea-sickness; and the too liberal use of wine. In almost every case in which I have employed it, complete relief has been the result; and that generally in less than two hours, and after three or four draughts.

In those cases in which the stomach is incommoded by offensive matter, it should be evacuated previously to the exhibition of the medicine. I have found that irritating the fauces has answered the purpose much better than giving emetics: in the greater number of cases, however, it will only be necessary to commence by giving

℞. Acetic acid, ʒj;
Compound tincture of cardamoms;
Simple syrup; of each, ℥ss;
Water, ℥x.

To be taken every twenty minutes, in the form of draught. One of the above draughts given early on the approach of an attack of headach, has often effectually warded it off.—*London Lancet.*

---

*On the mode of prevention of the Small Pox Pits.*—The importance of preventing the unseemly scars, consequent on small pox, will be a sufficient excuse for the trial of a practice, by several found successful.

The following plan, although known, has not been fully proved in this country; viz., the application to the affected parts of the "emplastrum ammoniaci cum hydrargyro."

We have taken some remarks from an inaugural thesis, read before the Faculty of Medicine of Paris, in 1840, by Dr. Joseph Olliffe, of Cork. The author first notices the "ectrotic" treatment (from the Greek εκτρωσις, signifying abortion, or destroyed before maturity.)

Bretonneau and Serres published the first papers on the subject, in the "Archives Generales," in 1825. The former pierced the point of each pustule with a gold or silver needle, armed with caustic, on the second or third day of the eruption.

Serres, in the confluent variety, painted the whole affected surface with a solution of the nitrate of silver; but in the distinct variety he merely touched each pustule.

M. Velpeau removed the top of each pustule entirely, and introduced the caustic into the cavity.

At the time this treatment created a great sensation, and was in general very successful; but it has fallen into disrepute, from its practice being in all cases painful, and in many impracticable: also, M. Guersent relates some cases where its practice was followed by cerebral affections; although Serres asserts that his plan prevents cephalitis, otitis, ophthalmia, &c., which so often follow small-pox.

Several other means have been recommended. "Cotugno" advised that the face should be washed frequently with warm milk and water.

The Arabians open the pustules, and press out the matter.

Dr. Picton, of New Orleans, excluded the light from the sick chamber, and in no one case were there any pits left.

M. Le Grand has recently read a paper before the Academy of Medicine, in which he states that the application of a thin layer of dissolved gum Arabic, to be immediately covered by gold-beater's leaf, prevents pitting.

Dr. M. Good recommends that a piece of fine linen, or cambric, on which some cetaceous cerate is spread, should be applied.

Mr. Wade has found benefit by using the solid nitrate of silver in the papular stage.

Mr. George also, from covering the pustules with calamine.

The method advocated by Dr. Olliffe is to mask the whole face with a sheet of the "unguentum ammoniaci cum hydrargyro," leaving orifices for the mouth, eyes, and nostrils: also, a small quantity of mercurial ointment is rubbed upon the eyelids.

Under this treatment the papules quickly disappear, or are converted into vesicles, or scabs. The fit period is in the papular stage, although even in the pustular stage it may be successful.

In the distinct variety the mask is kept on for three days, and in the confluent for four days. In reference to the *modus operandi* of the treatment, Dr. Olliffe asks, "Ought we to admit the existence of animalculæ?" and that they are destroyed by the mineral; or, "Does the metal act by diminishing the coagulating power of the blood?"

Before we can arrive at a knowledge of the *modus operandi* of the treatment, we must previously investigate the case of the scar, or pit. Mr. Ceely gives this explanation.

The eruption commences in papulæ, which have their seat in the corium. These papulæ consist of an adventitious membrane, formed in the corium, from a secretion by the papillæ. This membrane is raised in the form of a zone, and is intimately connected with the epidermis. When the pus forms, it separates the epidermis from the subjacent adventitious membrane.

When the pustules burst, or are broken, secondary inflammation is set up, the corium is destroyed, and the subjacent cellular tissue sloughs, leaving a deep red excavation, which forms the small pox pit.

We believe that the effect of the plaster is mechanical. It produces absorption of the peculiar secretion which forms the papulæ by an equable and continued pressure, and by a removal of the cause the secondary inflammation never occurs, and its ravages are thus prevented. That this is the *modus operandi* would seem to be proved by the fact, that the remedy loses its effect if not applied before the pustular stage. The mercury may have, besides, a specific effect, by stimulating the absorbing vessels.—*London Lancet.*

*Chemico-Legal researches on Morphia.*—Iodic acid has been offered as a test to indicate the presence of morphia; ss this has the property of depriving the acid of oxygen, as thus liberating iodine. Davidson denies the utility on this point, after the following observations:

While examining, some months since, the urine of a patient, he ascertained that on adding a portion of iodic acid, the urine acquired the odor of iodine; and starch with it produced an azure tint. I then examined, says he, what element of urine possessed this property. I tried both urea and uric acid, and discovered that the latter only produced this effect, and could thus be recognized in the urine of healthy persons, as well as in that of the sick. I afterwards examined the effects of iodic acid on the serum of blood, and obtained the same effects, but more slowly, and the colour was less deep than with the urine of persons in health. All the albuminous fluids which I examined gave the same results, the only difference being in the intensity of colour. I observed the same facts with regard to the serum of the brain of a man dead of typhoid fever, in the liquid evacuated by paracentesis after peritonitis, of ascites, in the serum of the milk of a heifer, &c. The serum of blood, even when mixed with four or five parts of water, gave, with iodic acid and starch, the same colour, but not so well as when undiluted.

It appears, from these facts, that iodic acid is susceptible of being decomposed by substances which exist in abundance, as an element of nutrition, and as a constituent of the liquids and solids of animals. One of the secretions, urine, contains a substance which has the same effect. Hence, on analysing the substances contained in the stomach, in a case of poisoning by opium, the certainty of finding albumen, and the possibility of meeting uric acid, can render the separation of the morphia from these two substances but very difficult and complicated, and even doubtful, so that iodic acid can no longer be considered as a test for Morphia.—*Journ. de Pharm.*

In a former number of the Examiner we published some interesting notices of the diseases of Peru by Dr. Archibald Smith. In the July number of the Edinburg Journal we find a continuation of these sketches, which will be no doubt as interesting to our readers as ourselves. The diseases of Peru are of course tropical, and therefore interesting to American readers, for the summer climate of a large portion of the United States is, as every one knows, a tropical one, the diseases are of course marked with many of the peculiar features of those of the torrid zone.

Dysentery is of course one of the most common diseases of the tropics; a large portion of Dr. Smith's remarks is, therefore, properly enough devoted to it.

*Practical Observations on the Diseases of Peru, described as they occur on the Coast and in the Sierra.* By ARCHIBALD SMITH, M. D.

DISEASES OF THE COAST.

*Dysentery.*—The nature of this disease, as it commonly occurs in Lima, where it may be said to be endemic, I shall endeavour to illustrate by a few remarks and examples,—arranging these under two general heads, viz. Simple and Complicated Dysentery.

The former embraces those cases in which the local irritation or inflammation, situated in the course of the great intestine, appears to constitute the primary and principal disease; the latter includes those cases of dysentery which obviously coexist with some other disease, or such cases as are dependent on, or complicated with, a morbid condition of the liver, either in structure or function.

*Simple Dysentery.*—The simplest form of dysentery in Lima is that which is known by the name of "Pujus blancos," or an urgent call to go to stool, while nothing is voided but white mucous matter. Here we have little or no fever, and no pain in the belly on pressure; the tongue is sometimes quite clean; but at other times it is foul, especially when the disease has lasted some days, and the pulse begins to rise.

This attack may come on suddenly from sleeping too lightly covered and exposed to a cold damp night air, or to a current from an open door; or it may have been preceded for several days by a sense of heaviness at the region of the stomach, which I have known induce a person so affected to visit the French hotel or Italian coffee-house, and there call for some nice dish to whet his impaired appetite, of which the consequence next day was tenesmus and white mucous dejections.

This sort of disease may be relieved by a purgative clyster followed by a sudorific, or a dose of syrup of morphia. I have seen it more than once removed by an emetic, which produced smart catharsis, and was followed by a curative sweat, favoured by diluents, and the warmth of bed-clothes. And here, by the way, it may be remarked, that the routine practice in Lima, of giving emetics in almost every case of dysentery, for three successive mornings after bleeding and purging, &c. have been premised, is highly objectionable in those instances where the liver appears to be the organ primarily and principally affected with increased sensibility or inflammation; as in many cases of complicated dysentery. But to return:—should the attack of simple dysentery, such as I have just described, be not speedily removed, it may assume a more serious form, and come under other treatment, as in the following example.

A young man continued to walk about the porticos of the great square to a late hour at night, and felt chilled before he left it. He retired to his quarters, went to bed, and next morning found himself troubled with tenesmus. Two or three days after he asked medical advice. I now saw him with some uneasiness at the hypogastrium, a soft skin, the tongue perfectly clean and moist, and of natural aspect; the pulse about 80°, full and soft; there was a good deal of tenesmus, and the scanty dejections were in appearance like white jelly, streaked with blood. He had taken no remedy before I was called to see him. I gave him a dose of castor oil, which afforded but little relief, and the ineffectual calls to ease the belly returned as before. He was then ordered five grains of extract of lettuce, and four grains of calomel every four hours, for a dose, and after it a glassful of linseed tea; and his diet was thin arrowroot. By this means constriction was removed, the bowels were opened, and gentle diaphoresis induced. After five or six doses of the calomel and extract of lettuce were taken, a dose of castor oil was given with excellent effect, and it dislodged freely the contents of the bowels. No salivation supervened; and by a few more days of restricted diet and care, health was completely restored.

A stout young gentleman from Europe, and not long resident in Lima, went to the Plaza on a warm morning, and took a glass of cold water; he then came home to breakfast, and, an hour after he had finished it, he felt wandering griping pains in the bowels; but these did not deter him from making a hearty meal at dinner. The griping pains became more frequent and molesting, and a few hours after dinner the pain became fixed in the lower belly. He took a dose of castor oil, and then went to walk in the streets and Plaza, thinking to shake off his indisposition by bodily exertion. He returned home at a late hour; laid himself down to sleep; passed an exceedingly bad night, with feverish heat, restlessness, headach, and a necessity to turn out of bed more than a dozen of times.

I saw him next morning, when his pulse was full and frequent, or about 90°; and he said that he was more serene at that time than he had been some hours before; for that the fever had subsided much, though he had no regular sweat, which he believed to have been checked by the frequent necessity of obeying the call of scanty and painful ejections. His tongue was white and dirty, the face flushed, and the pain in bowels distressing, with his mind anxious and disturbed.

His motions were now observed to be much tinged with blood. A nurse, who was ordered to attend him closely, applied an emollient cataplasm to the abdomen, and administered to him an emollient enema, with a view to moderate the tenesmus; and it returned charged with coagulated blood. He was then bled at the arm, and immediately after he took eighteen grains of calomel, mixed in a little gum Arabic and simple syrup. This dose quieted the tromina for a time, and soon after he voided two or three thick and spinage-like stools; and he again took smaller and repeated doses of calomel conjoined with opium, at proper intervals, till, on the third day from this attack, the gums began to be a little red and altered from their ordinary appearance, and therefore the calomel was suspended; and the patient was left to pass a day on diluents sweetened with syrup of ipecacuanha. By this means diaphoresis was promoted, and further time allowed for the absorption of the mercury, which was only beginning to develope its effects. Perspiration went and came occasionally, and partially, the tongue was yet foul, and the pulse down at 80. A purgative was administered with good effect: so that, having ceased to operate, the patient slept and perspired very freely in the course of the night. On the morning after (being the fifth day of the complaint) the bowels were thus efficiently moved, the patient for the first time complained of his mouth, and, on inspection, the gums were seen to be red, well injected with blood, full and tender, and the palate looked risen, corrugated, and somewhat blistered; but no ptyalism was present, though the mouth and tongue were humid. The tenesmus disappeared; the urine continued to be a little too highly coloured; but no pain or uneasiness on pressure remained in the abdomen. He now perspired continually, and the pulse became soft, and of natural frequency. His spirits grew buoyant; he felt well, and, if permitted, would have risen and dressed himself. He used arrow-root diet, as he did from the commencement, and drank linseed tea as often as he liked.

On the sixth day from the invasion, he avoided, without the aid of opening medicine, a perfectly natural looking deposition; and from this date his convalesence was rapid, and his hunger so craving, that he was importunate for better and more plentiful diet than could be prudently allowed till some days more had elapsed, when he went on a visit to the country. He soon returned to his counting-house in good and vigorous health.

A yet more common form of simple dysentery than either of the preceding is, when the pulse becomes febrile, though not very much so; when the tongue is dirty, though moist; the griping and tenesmus troublesome, but not excessive, with little or no tenderness in the abdomen; the motions scanty, and consisting chiefly of mucous and bloody matter, more or less intimately mingled together, in varying proportions.

The practice which I found invariably successful in such cases, which are of frequent occurrence, was at the first visit to order a suitable purgative, such as the infusion of senna, with the addition of manna and rhubarb, or a dose of castor oil. The purgative usually carried off a portion of green or yellow liquid discharge, but seldom any considerable portion of feculent matter, and the stools came away, for the most part, as if they had been strained or filtered through the contracted part of the intestine. After the action of the purgative, there were given night and morning, five grains of calomel, with one grain, or one-half grain of opium, combined in form of pill; but every other morning this remedy was not given till the intestines had been previously acted upon by a suitable clyster or mild purgative. On the fourth or fifth day of this treatment, the gums were affected, and then feces, commonly of a dark colour, and offensive in odour, were voided, and not rarely several black gelatinous-like stools came away. These jellied motions were always, when they appeared, viewed as favourable indications of the mercurial action, and of returning health. The secretions now became improved, and the perspiration free; the tongue gradually got clean; the appetite was good; the powers of digestion grew vigorous; the motions assumed a natural appearance, and the patient did well and soon recovered. The soldier in hospital, when thus treated, was usually returned fit for duty, in less than twenty days from the time of his admission.

I feel gratified in being able to say that my friend, Dr. Maclean, who succeeded me in the charge of British and American seamen and subjects in the hospital of San Andres, Lima, assured me, just before my departure from that city, that, during the few weeks he had been attending at the request of the director of the Public Beneficencia, some of the Indian soldiers in the medical wards of this hospital, he treated not less than fifteen or sixteen cases of dysentery without having once had occasion to use the lancet; that he cured all of them with calomel; that it was quite extraordinary how certain the remedy proved; for that no sooner was the mouth gently touched with the mercury than the secretions improved, gentle perspiration was induced, and health followed.

Though I have always observed that a warm and general diaphoresis, or a more profuse sweat, proved a salutary process, resulting from mercurialization in dysentery, yet I do not believe that the salutary effects of the mercury depended on its sudorific powers alone, (because simple sudorifics have not the same curative efficacy,) nor yet upon the revulsion which it occasions through the salivary organs, because, in general, ptyalism, either not at all, or only in a very moderate degree, was found essential to the production of the salutary change induced by mercurialization. In well established and developed forms of dysentery, sudorifics may be useful auxiliaries, and the soothing combination of calomel with opium has a well known sudorific effect. But, from the frequently repeated connection which I have had the opportunity to observe between the point of mercurialization, indicated by slight affections of the gums, and returning health, I conceive that mercury has a peculiar and extensive influence on the capillary vessels, which serves to dissolve the inflammatory action in dysentery, and to render it one of our surest remedies in most cases of this severe disease. In the treatment of simple dysentery I have been in general very sparing of blood-letting; and chiefly for this reason, that on the Peruvian coast, the organs of digestion are often in a feeble state, and consequently the repair of the system, after its strength is reduced by blood-letting, is slow and sometimes imperfect; yet, in cases of high excitement, increased sensibility in the belly when pressed upon, or when the motions contained much pure blood, in a recent and acute attack of this formidable ailment, I have used the lancet pretty freely to lower excessive vascular action, or remove a dangerous degree of congestion; and thus have prepared my patient for the safe and efficacious action of mercury. But in some cases, when the stools contain much blood, and are, at the same time, accompanied with unusual chillness and general depression, bleeding may be dispensed with, as in the following case.

A soldier in hospital was in hot weather affected with severe tenesmus, and his stools were almost pure blood; the pulse was depressed; the tongue was yellow and furred; the lower extremities and belly were cold, with slight occasional perspiration confined to head and neck. He was put at once on a diluent and farinaceous diet; took eight grains of calomel and one of opium, morning and evening; emollient cataplasms and lavements were duly employed, and on the fourth day of this treatment, his mouth appeared gently affected. This indication of mercurial influence was followed by gentle but general warm diaphoresis. Griping pains and tenesmus ceased; the patient voided yellow feculent stools; and there was no longer any appearance of blood in the motions. I may add that while in this convalescent state, the patient was seized with an aguish fit. He was kept on diluents for a couple of days, on each of which there was manifested a well-formed paroxysm of quotidian ague. He took opening medicine, which acted freely, and carried off motions of a yellow appearance. On the third day of the fever he took quinine, and in two days more, the intermittent was cured, and the patient did well.

This complicated case shows that blood-letting may sometimes be very safely and properly avoided, even though the character of the dejections be highly sanguineous. The precedency, however, due to the lancet under the opposite circumstances, of local pain with general excitement, is the more worthy of attention, since it is a fact, that, as mercury begins to show its effects, or as it approaches that point of saturation indicated by a corresponding change in the condition of the salivary organs, it increases the frequency and force of the pulse before mild ptyalism is established, or before the constriction of the intestines is relaxed, and the condition of the extreme vessels or capillary system altered. But, should febrile action happen to be considerable before mercurialization is resorted to, even without the previous subtraction of blood, no sooner is the system fairly brought under its influence than the consequent opening of all the sluices, perspiration, exhalation, and secretion, co-operate in reducing the pulse and moderating or removing every bad symptom. Knowing such to have been the usual beneficial operation of mercurialization in numerous cases that came under my own superintendence, I think it is correctly stated by a modern writer,* that "mercurial preparations generally possess in a higher degree than any other known medicine the power of changing the condition of action in the extreme vessels of the circulating system throughout. It is for this reason that it is so important an instrument in the hands of the physician in so many and apparently dissimilar complaints; and it is proved by experience to exert no greater influence over the secreting vessels of the liver than it does over those of the intestines and mesentery."—This is a matter of fact and experiment, which all who have the opportunity of observation and practice in the delicate climate of the Peruvian coast, to which my observations more particularly refer, may repeat and verify, for their own satisfaction and the good of the sick. And here I may be allowed to offer an opportune warning, viz. that the patient, especially if a delicate Limenian, should avoid drinking cold water, or cooling beverages, or exposure to a current of cool air, while under the influence of mercury, lest the cutaneous secretion should be suppressed, and the salutary action of this remedy disturbed, or its effects too much concentrated on the bowels and internal organs.

I think myself warranted by experience in remarking, that in negroes the system is not so easily brought under the influence of mercury as in Indians and Europeans. In elderly people, and in those of broken down constitutions, the capillary vessels appear to be less capable of taking on a healthy and vigorous mercurial action, and for such persons the mercurial treatment is of course less appropriate. But the Indian, recently arrived from the mountains, who is seized with dysentery on the coast, is more susceptible of an easy cure of this complaint by mercurialization, than many among the more delicately framed and dissolute Limenians, in whom the vascular, like the muscular system, is comparatively weak and lax, while the nervous system, likewise, is in those addicted to debauchery less sound and vigorous. And though it is objected to mercury, that its effects on the nervous system are pernicious, especially when injudiciously employed, or too long continued, these evil consequences are, perhaps, to a still greater extent, chargeable to the account of many other useful remedies, not even excepting that indispensable drug, opium; for the excessive use or abuse of this and other medicinal agents from the vegetable kingdom, produces nervous debility, and exhausts the vital powers. We must, therefore, continue to use mercury in a variety of diseases, not excepting dysentery, till we find a less exceptionable substitute; and it is no small recommendation of this remedy, that, if its good effects are not always complete in Peruvian dysentery, they are at least predominant. I do not say that the plan which is here recommended, and which I myself, as well as several other foreign, and even native practitioners in Lima, have adopted in public, and, as often as circumstances did permit, in private practice, is the only good curative method in all such cases of dysentery, as those in which we found it deserving our greatest confidence. But, taking impartial observation, and ample practical experience of the fact, as the foundation of my own convictions on this subject, I can confidently and conscientiously bear witness that mercurializing the person affected with dysentery, in the manner which I have described, (and as this disease most usually presents itself on the coast and in the intermediate valleys of Peru,) is really safe and good practice, and when properly conducted, it will, I am persuaded, be more generally crowned with success than any other plan of treatment which I have found recommended in books, or seen applied in usual practice.

Regarding the administration of mercurial remedies, in general, in this disease, and of calomel, in particular, I observe, that, though in general, it be a sovereign remedy in the hands of those who know how to use it discreetly, in such common cases as I have taken pains to characterize, just as bark is known to be a very superior remedy in ordinary cases of ague; yet it is no more an infallibly specific remedy in dysentery than bleeding is in every case of inflammation of the lungs; nor does its administration necessarily supersede the co-operation of other means of relief; and there are, moreover, circumstances under which its exhibition is fruitless and inexpedient. When we witness a person affected by cold and partial sweats with hiccup, or fever and general

* See Graham on Indigestion.

sweats without any corresponding alleviation in the dysenteric symptoms; when the stools look like flesh-washings, without the appearance of mucous, feculent, or bilious matter; when the inflammation, no longer confined to the lower intestine, extends over the neighbouring viscera, and the belly becomes elevated and acutely sensible to pressure; when such is the state of the patient, even should he have been previously bathed and bled till he can hardly bleed any more; should he, in the hands of the consultation, have passed through all the routine of emetics, ptisans, chicken-soup, clysters, oils, cawls, plasters, blisters, and sinapisms, we may freely confess that, under these calamitous circumstances, calomel is a remedy which we would never propose or recommend. In such extreme cases, the membranous texture of the diseased intestines is too extensively disorganized, and the capillary vessels are no longer able to take upon them a sanative mercurial action. Cold extremities, a thready or imperceptible pulse, and relaxation of the *sphincter ani*, indicate that death is, in most such instances, very near at hand. In so deplorable a state, the Peruvian patient is aided by the priest, and his doctor prescribes clysters of the decoction or infusion of bark, or orders ice inwardly as the last mean, when, to another person's touch, the apparently moribund patient outwardly feels as cold as the iced-water he drinks; and when the feeble powers of ebbing life are concentrated, without being able to rally again from their last retreat in the internal recesses of the body.

It is not, I think, in these circumstances, much discredit to phlebotomy, calomel, or any other principal remedy, to say that it offers but little chance for recalling vitality and circulation to the surface and extremities, or energy and health to the general system. But, in conditions apparently the most hopeless, the female practitioners follow the physician, and really can boast of restoring a few to health, by herbs and nostrums of their own unpublished Materia Medica, which the quack doctress carefully conceals from the scrutiny of the regular doctor, and curiosity of the protomedical tribunal, inimical to her fame, and to the alleged abuses of her wonder-working xeringa or clyster syringe.

While offering these remarks on this important subject, I should not omit to mention certain diversities in the mode of curing dysentery, which several French individuals resident in Lima adopt for themselves or recommend to others. For example, a commercial gentleman, who had long suffered from a dysenteric attack, of which he had thirteen relapses, was at length told by his native medical attendant, that he had no remedy left him to be tried, unless, as a last resource, he would submit to take calomel. To this proposal, the patient replied, calomel is an English remedy; but I am a Frenchman. I wont have it. I therefore, says this gentleman, took my own way of it. Observing that when in best health it used to cost me much trouble to digest two meals a-day, I resolved in my sickness to subject myself to one meal only, which consisted of rice plain boiled in water, with a trifle of salt to it. About eight hours after this meal, the stomach used to feel empty, and then I drank a glass of lemonade, and afterwards took some of this beverage as often as I felt inclination, especially during night. Every three or four days, lest there should be any accumulation in the bowels, I took a little cream of tartar in my first or morning draught of lemonade. My rice diet, with little variation, was continued for one year, and after this manner I cured myself and many others.

Betwixt this patient's treatment of himself and that pursued by a countryman of his own, —a scientific dentist in Lima,—for a dysentery he also became subject to, there was a wonderful contrast; though in the latter case the result was equally successful, and the end much sooner attained. The dentist's pulse was about 80; the tongue very foul ; tenesmus most troublesome, and attended with slimy dejections, but little or no appearance of blood, and he had no pain in the belly. He cured himself with the famous panquemagoga of Le Roy, taken according to the usually prescribed method.

The mercurial practice in simple acute dysentery is, we know full well, the most certain of success. Our own method of administering the remedy differs from that followed by Dr. Smith, and many other practitioners. We give calomel or sometimes blue mass, or the hydrargyrum cum creta in smaller doses, so as to affect the system gradually, and, after the lapse of two or three days, by continuing the mercurial with ipecacuanha and opium, the patient is immediately relieved of some of the most distressing symptoms, and is in a great degree tranquilized, while the ultimate action of the mercurial is not interfered with. The beneficial influence of mercury is not equally certain in different years and epidemics of dysentery, probably because the disease is not always equally inflammatory, and mercury is much better adapted to this variety than to any other.

---

*Galvanism.* By Richard Fowler, M.D.—I have seen in the Gardener's Magazine for the last month, (April, 1841,) an advertisement of a "new discovery," (a galvanic plant protector,) formed of broad bands of zinc and copper, so placed in relation to each other and the stems of plants, that snails and caterpillars attempting to crawl over them with their moist surfaces are intercepted by the metallic sensation, probably similar to that which is felt on the tongue when in con'act with zinc and sil-

The experiment which suggested this ingenious application of zinc and copper is thus stated, page 225, Gardener's Magazine for April:

"If a snail or slug be placed on a plate of zinc, to which a narrow plate or strip of copper is fixed near the edge, and the zinc turned over it so as to form a ring of zinc, copper, and zinc, it creeps unmolested on its surface. But as soon as it touches the rim where the copper is, it receives a galvanic shock, its moistened soft body, acting as moist cloth (between plates of zinc and copper in a pile,) and thus forming the galvanic circle complete, and immediately recoils, twisting itself back, and rarely venturing a second time to touch the copper, to receive another shock."

In the year 1793, while yet an undergraduate in the University of Edinburgh, I published the following experiment, with many others, on the nerves, senses, and muscles:

"I had laid a leech on a crown piece of silver, placed in the middle of a large plate of zinc. The animal moved its mouth over the surface of the silver, without expressing the least uneasiness; but having stretched beyond it, and touched the zinc plate with its mouth, it instantly recoiled, as if in the most acute pain, and continued thus alternately touching and recoiling from the zinc, till it had the appearance of being quite fatigued. When placed wholly on the zinc it seemed perfectly at ease; but when its mouth came in contact with the silver lying on the zinc, the same expression of pain was exhibited as before. With the earth-worm this experiment succeeded still more decidedly; the animal sprang from the zinc in writhing convulsions. If, when the worm stretched itself forwards, one of its folds lit upon the zinc, it expressed little uneasiness in comparison of what it showed when the point of its head touched the zinc. These extraordinary effects were, however, considerably different from those produced by the metals on the limbs of frogs and other animals. They had not so much the appearance of involuntary instantaneous convulsions, as long continued expressions of pain and disgust, such as are produced by applying zinc and silver to the tongue of a child: a strong presumptive proof, in my opinion, that these animals are endowed with a most exquisite organ of sense; and consequently not, as had been supposed, destitute of a system of nerves."

I have been so much out of the way of all literary transactions for nearly half a century, that I am afraid I may be making an unreasonable request in begging the favor of you to publish this unvarnished statement of facts in your very correct and impartial journal.

To the useful application of the experiment I can have no claim; nor should I trouble you with this proof of priority of observation of its results, were it not one of a series in an investigation of phenomena connected with the fifth pair of nerves.

Some part of this subject I communicated to Section E, of the British Association, at Glasgow, last autumn, a short notice of which may be found in the volume of Reports, just published.

Nearly the whole of the experiments alluded to were repeated in the presence of gentlemen yet alive, and who still permit me to call them friends: Mr. Allen, Master of Dulwich College, Professor John Thomson of Edinburg, and Dr. A. Monro. As I was at that time a pupil of the late Mr. John Bell, the experiments were probably seen also by Sir Charles Bell. The experiments succeed best when snails, worms, and metals, are wet with rain water.

Far be it from me to impute intentional plagiarism to the author of this clever application of an experiment so similar to mine, made in 1793. Nothing is more likely to happen than that men not in communication with each other, but occupied in similar investigations, should adopt like means, and arrive at like results.

Professor John Robinson, a lecturer on natural philosophy in 1793, sent me a letter, of which I inclose you a copy. As it may interest those who have not met with it, to see how near Professor Robinson was to the dis covery of the pile, which immortalized Volta, you may perhaps think it worth while to republish the letter.

*To Mr. Fowler.*

EDINBURG, May 28, 1793.

Sir,—About a fortnight ago, my son told me of a curious experiment, with a piece of zinc and a piece of silver applied to the tongue, by which a strong irritation, resembling taste, was produced, and that a luminous flash was excited, by applying one of them to the eye. I immediately repeated them according to his directions, and my curiosity was greatly excited to prosecute them in a variety of circumstances. I understand that these experiments have originated from the curious discoveries made some time ago in Italy, of which I was informed last winter. But I have been so much out of the world for some years past, that I have had no opportunity of knowing what was going forward.

Being informed, that you have been long engaged in experiments on this subject, and are about to favor the public with an account of them, I have taken the liberty of communicating to you a few facts which have occurred to me, some of which, perhaps, may be new to you.

1. I find, that if a piece of zinc be applied to the tongue, and be in contact with a piece of silver, which touches any part of the lining of the mouth, nostrils, ear, urethra, or anus, the sensation resembling taste is felt on the tongue. If the experiment be inverted, by applying the silver to the tongue, the irritation produced by the zinc is not sensible, except in the mouth and urethra, and is very slight. I find the irritation by the zinc strongest when the contact is very slight, and confined to a narrow space, and when the contact of the silver is very extensive, as when the tongue is applied to the cavity of a silver spoon. When the zinc touches an extensive surface, the irritation produced by a narrow contact of the silver is very distinct, especially on the upper side of the tongue, and along its margin. This irritation seems to be mere pungency, without any resemblance to taste, and it leaves a lasting impression, like that made by caustic alkau.

2. If the zinc (finely polished) be applied to the ball of the eye, the brightness of the flash seems to correspond with the surface of the contact of the silver with the tongue, palate, fauces or cheek. The same thing happens when the silver is applied to the eye.

3. When a rod of zinc, and one of silver, are applied to the roof of the mouth, as far back as possible, the irritations produced by bringing their outer ends into contact, are very strong, and that by the zinc resembles taste, in the same manner as when applied to the tongue.

4. I had been paring my toe nails with scissars, and had cut off a considerable portion of the thick skin, so that the blood began to ooze through, in the middle of the wound. I applied the zinc there, and an extensive surface of silver to the tongue. Every time I brought the metals into contact, I felt a very smart irritation by the zinc at the wound.

5. I made a piece of zinc having a sharp point, projecting laterally from its end. I applied this point to a hole in a tooth, which has sometimes ached a little, and applied the silver in an extensive surface to the inside of the cheek. When the metals were brought into contact, I felt a very smart and painful twitch in the tooth, perfectly resembling a twitch of the toothache. I thought this twitch double, and that one of them happened before the metals came into absolute contact. I am now almost convinced that this is the case, for when I make the silver rest on a dry tooth, without touching the tongue or fauces, I have no twitch on bringing the outer ends of the metals together: showing that there is not a proper communication through a dry tooth. If, while the outer ends remain in contact, I touch the silver with the tip of the tongue, still no twitch is felt in the tooth. If I now separate the outer ends of the metals, keeping the tongue applied to the silver, a slight twitch is felt in the moment of separation, when they are again brought into contact.

N. B. This twitch is prevented, by allowing the tongue or lip to touch any part of the zinc.

6. I had a number of pieces of zinc made of the size of a shilling, and made them up into a rouleau, with as many shillings. I find that this alternation, in some circumstances, increases considerably the irritation, and expect, on some such principle, to produce a still greater increase. If the side of the rouleau be applied to the tongue, so that all the pieces are touched by it, the irritation is very strong and disagreeable. This explains what I have often observed, the strong taste of soldered seams of metal. I can now perceive seams in brass and copper vessels by the tongue, which the eye cannot discover, and can distinguish the base mixtures which abound in gold end silver trinkets.

If any of the above facts can add to the stock of knowledge you have acquired on this subject, it will give me great satisfaction, and I shall not fail to communicate any thing which may afterwards occur. My indisposition hinders me from taking an active part in the researches, to which this wonderful and important discovery incites; but it is both my duty and my earnest wish, to contribute my feeble assistance to every gentleman engaged in this interesting pursuit.

I find that common silver thread makes a very good conductor, and this to any distance.

Since writing the above, I have found a very easy way of producing very sensible convulsions (I think muscular) and corroborating my opinion, that the communication (of this part of the whole effect) takes place before contact.

Put a plate of zinc into one cheek, and a plate of silver (a crown piece,) into the other, at a little distance from each. Apply the cheeks to them as extensively as possible. Thrust in a rod of zinc between the zinc and the cheek, and a rod of silver between the silver and the other cheek. Bring their outer ends slowly into contact, and a smart convulsive twitch will be felt in the parts of the gums situated between them, accompanied by bright flashes in the eyes. And these will be distinctly perceived before contact, and a second time on separating the ends of the rods, or when they have again attained what may be called the striking distance. If the rods be alternated, no effect whatever is produced.

Care must be taken not to press the pieces hard to the gums; this either hinders us from perceiving the convulsion, or prevents it. I find, too, that one rod, whether zinc or silver, is sufficient for the communication; and even bringing the two pieces together will do as well, or perhaps better. But the rods are easier in the management.

Asking pardon for the liberty I have taken, without having the honour of your acquaintance, I am, with great regard, sir,

Your most obedient humble servant,

JOHN ROBISON.

*Lond. Med. Gaz.*

